# Prevalence and predictors of HIV sero-discordance among cohabiting couples tested in northern Tanzania

**DOI:** 10.11604/pamj.2015.22.275.5961

**Published:** 2015-11-23

**Authors:** David Paul Ngilangwa, Rhoune Ochako, Beati Alphonce Mboya, Rita Honoratha Noronha, George Suleman Mgomella

**Affiliations:** 1 Amref Health Africa, P.O Box 2773 Dar es Salaam, Tanzania; 2 Kilimanjaro Reproductive Health Program, Moshi, Tanzania; 3 Population Services International, Nairobi, Kenya; 4 Department of Medicine, Strangeways Research Laboratory, University of Cambridge, Worts’ Causeway, Cambridge CB1 8RN, UK

**Keywords:** HIV, prevalence, sero-discordance, cohabiting couples, Tanzania

## Abstract

**Introduction:**

In sub-Saharan Africa where HIV/AIDS epidemic is predominantly generalized, majority of HIV infections occur among heterosexual couples. The majority of people do not know their sero-status. Thus, utilisation of Couples’ HIV Counselling and Testing (CHCT) services remain to be critical in preventing new infections. The objective was to establish prevalence and predictors of HIV sero-discordance among cohabiting couples presenting for CHCT services in northern Tanzania.

**Methods:**

A cross-sectional study inteveviewed 1,333 couples aged 18-49 years tested from 2005 to 2007 in Kilimanjaro and Arusha regions. A CHCT checklist was used to collect data from couples. Data were analyzed using STATA 10.

**Results:**

Generally, 220(16%) out of 1,333 couples were HIV sero-discordant. In sero-discordance unions, women were likely to be HIV positive than men (71% versus 29% respectively p<0.001). HIV sero-discordant relationship was associated with age (35-45 years) for both men and women (Adjusted Odds Ratio (AOR): 2.3, 95% Confidence Interval (CI): 1.7-3.2) and (AOR: 2.6, 95% CI 1.9-3.7) respectively. Women with older men partners were less likely to be in HIV sero-discordance relationships (AOR: 0.5 95% CI 0.3-09). Arusha couples were likely to be HIV sero-discordant than those of Kilimanjaro (AOR: 2.3 95% CI 1.7-3.2). Couples living far away from CHCT centres were less likely to be sero-discordant than those live nearby (AOR: 0.4 95% CI 0.2-0.9).

**Conclusion:**

HIV sero-discordance prevalence is high among our participants. Thus, we recommend CHCT utilization should widely be promoted as entry point in treatment as prevention strategy in order to protect uninfected partners in HIV sero-discordance relationships.

## Introduction

Globally, in 2013, 2.1 million and 1.5 million people were HIV newly infected and died from AIDS-related complications respectively. However, massive investments to curb HIV/AIDS epidemic have led to reduction of burden in some parts of the world [1, 2]. Yet, Sub-Saharan Africa (SSA) accounts for 70% of the global burden of the epidemic. Heterosexual sex remains to be the predominant mode of HIV transmission in SSA[1].

Most of the countries in the region are experiencing a generalized epidemic whereby infections are occurring among adults with stable and long-term sexual relationships-specifically cohabiting couples [3, 4]. HIV sero-discordance-one partner is HIV infected while the other one is not-accounts for 75% of all HIV infected couples aged between 20-49 years in low prevalence (<10%), on ther hand it only affects and 50% in high prevalence (>10%) countries respectively [5–7]. Recent evidence from SSA shows that stable HIV sero-discordant couples contribute 30% of new HIV infections transmission from the infected to the uninfected partner within the couple [8]. Tanzanian HIV and AIDS population-based survey showed that among the couples in current union, 93% were HIV concordant negative, 2% were HIV concordant positive while 5% were HIV discordant; whereas in 3% of sero-discordant relationships the men partner was infected and the women was not [9]. In most instances, proportion of infected men and women in these relationships are almost similar [10].

Evidence from studies carried across the region suggests that early sexual debut, concurrent partnerships and socio-cultural practices that encourage men to engage in extra marital relationships are risks factors for HIV sero-discordance before and during cohabitation [1, 9, 11, 12]. Living in HIV sero-discordance relationships increase the odds of HIV negative partner being infected by HIV by 8-26% yearly as compared to compared to those who live in HIV concordant negative couples [11, 13]. The risks of HIV infection in sero-discordance relationships increase when the regular partner is newly infected as well as when there is a desire to have children and therefore condom use during sex is rarely [6, 13–17]. Moreover, a multi-country study conducted in seven African countries among 3,408 sero-discordant couples found a high rate of mortality attributable to HIV infection [18].

Majority of sero-discordant couples are unaware of their sero-status or presume to have the same status as that of their partners *(HIV testing by proxy)*. This is either mainly due to the fact that they rarely are tested or test individually anddiscloses statutes or not to their partners [19]. Findings from nationally representative population-based surveys in several SSA countries showed that more than three-quarters of stable couples with at least one HIV-1-infected partner were unaware of their partner′s HIV status [6, 20]. In such situation, advocating for ‘B’ (be faithful) among couples could be a risk for infection unless both partners know their status and agree to protect each other [21].

Couple HIV Counselling and Testing (CHTC) continues to be an important strategy for sero-discordant couples to access HIV treatment as prevention, whereby infected partner are put in antiretroviral therapy(ART) immediately after testing [20, 22]. Besides, when partners learn their HIV status together challenges related to stigma, violence, divorce and abandonment are automatically get addressed [6, 23–28]. Also, evidence from different SSA countries confirms effectiveness of CHCT in increasing condom use among HIV sero-discordant couples [29], nevertheless, women partners’ inability to negotiate, misconceptions about HIV-1 sero-discordance, and desire for children continue to impede its consistence use [30, 31]. However, studies in Tanzania and Zambia have shown low acceptability by men when CHCT services are incorporated within existing healthcare services like Antenatal Care (ANC) [32, 33]. Consequently, developing freestanding CHCT centres where spouses can be tested and learn their status together becomes vital to address disclosure related challenges and low utilization of services [3, 7, 21, 34–37].

In Tanzania, the uptake of HIV testing and counselling services among sexual active individuals aged 15-49 years had been increasing. For instance, from 2007-2011 proportion of women and men who knew their HIV status went up from 41% and 29% to 62% and 47% respectively [9, 12]. Among the factors attributed to this achievement is the implementation of a national HIV counselling and testing campaign led by the President of Tanzania from 2007-2008 which attracted about 4.8 million to get tested and wide coverage of HIV testing outlets countrywide [9, 38–40]. However, Tanzania like many other countries with generalized HIV epidemic in SSA had neither implemented extensively provision of CHCT services nor documented the prevalence and predictors of HIV among sero-discordant couples seeking for services [7, 9, 12, 13, 35, 41, 42].

This study aims to establish prevalence and predictors of HIV sero-discordance among couples and the uptake and utilization of CHCT services presenting for testing in two towns of Moshi and Arusha in northern Tanzania.

## Methods

### Study design, population and settings

This was a cross-sectional study of all couples aged between 18-49 years, who sought CHCT services in stand-alone centres established by Kilimanjaro Reproductive Health Program (KRHP) in Kiusa Street in Moshi municipality and Clock Tower in Arusha municipality from June 2005 to April 2007.

KRHP was a collaborative research program of Kilimanjaro Christian Medical Centre (KCMC), a teaching hospital in Moshi, Tanzania and Harvard T.H Chan School of Public Health (HSPH) of Massachusetts, in the United States. The programme conducted numerous HIV prevention randomized clinical trials within the study catchment. The two stand-alone CHTC clinics were established in efforts to identify couples which were heterosexual HIV-1 sero-discordant for enrolment into the Partners in Prevention HSV/HIV Transmission Study, details of which have been published elsewhere [43].

In promoting uptake of CHCT services numerous promotion strategies including radio, street banners, posters, leaflets, public address system in different gatherings and outreach staff were used. In addition, all motivated couples who came and received services were asked to invite other couples as well. The services were offered free of charge for six days a week except on Sunday, when necessary special arrangements were made to test couples during Sundays and public holidays. Furthermore, we collaborated with four Voluntary Counselling and Testing (VCT) centre, two from Moshi and two from Arusha, to provide outreach services. Data of couples from these four VCT centres were not included in analysis of this study.

### Data collection and counselling procedures

Trained study counsellors collected socio-demographic information of couples presented for testing using pre-tested clinic checklists. The following information was collected: age, area of residence and source of CHCT information that motivated couple to come for testing, HIV testing history and HIV test results. The process of collecting information lasted for about 10 minutes. To ensure quality of data, trained CHCT clinic supervisors and study coordinator reviewed all the collected data on the daily basis. Counselling and testing was conducted according to WHO's guidance which requires both men and women partners to receive counselling and HIV test results together, then share their results confidentially [22].

### Laboratory procedures

Couples were invited for venopuncture after CHCT. Blood spots samples for each participant were drawn from the vein by trained counsellors who were mainly registered nurses/midwives. Then blood spots were tested in the clinics’ laboratories simultaneously using two tests, the Determine^®^ HIV/2 test (Abbott Diagnostic Division, Hoofddorp, The Netherlands) and the Uni-Gold HIV test^®^ (Trinity Biotech^®^, Dublin, Ireland). Samples with indeterminate or discordant results between the two rapid tests w confirmed with enzyme-linked immunosorbent assay (ELISA) at the Department of Clinical Laboratory at the KCMC.

### HIV status information

HIV statuses of couples were filled in on the clinic interviewer administered checklists after completion of testing process. The key goal of promoting CHCT was to recruit HIV sero-discordant couples for randomized clinical trial to test the efficacy of twice daily acyclovir 400 mg given to the HIV-infected partner to prevent transmission to the HIV negative partner(s); its results has been reported elsewhere [43]. Therefore, HIV sero-discordant couples were asked to undergo further screening procedures for enrolment in the main clinical study [43]. However, those who were not interested to join the study were referred to the nearest HIV Care and Treatment Centre (CTC) at either KCMC or Mount Meru hospital. Moreover, all HIV concordant positive couples were referred to the national CTC outlets of their choice or convenience. HIV concordant negative couples received HIV and STI prevention counselling and were encouraged to keep on attending CHCT regularly.

#### Ethical consideration

Ethical clearance for this study was obtained from ethical committees of Kilimanjaro Christian Medical College, Harvard T.H. Chan School of Public Health, University of Washington and the National Institute of Medical Research of Tanzania. All participants in this research volunteered to take part after being explained to, the benefits, risks, confidentiality and anonymity of information they were going to give. All the information collected from couples was kept in confidential and instead of using their names, couples were given unique numbers to be used in analysis.

#### Data processing and analysis

##### Data entry

Couples clinic checklists information were re-evaluated for their completeness and any inconsistencies rectified on daily basis. The coded clinic checklists were double entered in EPI Info version 3.3.2(Centers for Disease Control and Prevention, 2001) and stored in the MS Access 2003 database. Subsequently, we conducted a random check of 10 per cent of the entries in the database by removing outliers.

##### Data analysis

All data analyses were done by using the statistical software Stata version 10 (Stata Corporation, College Station, Texas, USA). Couples data were categorized according to their sero-status as concordant negative (HIV-, HIV-), concordant positive (HIV+, HIV+) or HIV sero-discordant (HIV+, HIV-). Socio-economic status of couples where categorized with regards to reported place of residence affluent areas being highest and deprived areas being the lowest. The same was done for the middle class.

Descriptive statistical analyses was thereafter done to obtain HIV prevalence and distribution of couples according to their socio-demographic characteristics like age, partners’ age difference, area of residence and socio-economic status; frequently reported source of information for coming to get tested. Bivariate analyses were conducted to find associations between couples’ HIV sero-status against demographic factors. The associations across categorical variables was measured by Chi-square with corresponding p-value, the same tests were done for source of information. Groups with a sample size of ≤5 were compared using Fisher′s exact test. The age differences by gender were assessed using Student′s t tests. P-value of less than 0.05 was considered as statistically significant.

Logistic regression model was fitted to assess predictors of HIV infection or living in HIV concordant positive or discordant relationships. The same model was fitted for the predictors of reporting particular source of information among infected couples. Additionally, confounding factors were controlled for by performing multivariate analysis and including only variables that were statistically significant at 95% (p<0.05) and 90% (P<0.10) in the univariate logistic analysis by adjusting for all variables. Odds ratios (OR)-crude and adjusted-are reported with 95% Confidence Intervals.

## Results

### Demographic characteristics of the study participants

A total of 1333 (95.2%) out of 1400 couples were eligible to participate in this study. Their mean age was (31.63 ± 9.53) years. Mean age for men partners was slightly higher (34.6 ± 9.89) compared to that of woman counterparts (28.8 ± 8.25). This difference was statistically significant (*t*-test, 95% Confidence Interval 4.93-6.31, p <0.001). Majority of the couples1, 153 (86.5%) had men partners slightly older as compared to their women partners whereby, among 111(8.3%) couples, women were older. Only 69(5.2%) of the couples had each partner of a similar age.

More than half, 690(52%) of the participants were tested in Clock Tower CHCT centre in Arusha. Similarly, about 52% of the tested couples reported to be residing in Arusha region while and 47% from Kilimanjaro region. Only 11(1%) of tested couples were from neighbouring towns other than Moshi and Arusha. Most, 862 (65%) of the participants were residing within 4 kilometres from the CHCT centres. Majority, 947(71%) of the participants were from urban areas, followed by rural 360(27%) and only 26(2%) were from semi-urban areas. Characteristics of the participants are shown in Table 1.

**Table 1:** Characteristics of couples tested in Moshi and Arusha 2005-7

Variable	Total number (N)	Per cent (%)
**Men (age in years)**		
<19	4	0.3
20-24	166	12.4
25-29	337	25.3
30-34	263	20.0
≥35	563	42.0
**Women (age in years)**		
<19	118	8.9
20-24	351	26.3
25-29	342	25.7
30-34	226	17.0
≥35	296	22.2
**Age difference within the coupe in years**		
M<F∞	111	8.3
M=F¤	69	5.2
1 to 5	566	42.5
6 to10	368	27.6
≥11	219	16.4
**Socio-economic status**		
Low status	440	33.0
Medium status	803	60.2
High status	90	6.8
**Distance from home to the testing centre (in kilometres)**		
0 to 5	862	64.7
6 to 10	78	5.9
11 to 15	122	9.2
16 to 20	64	4.8
≥21	207	15.5
**Nature of place of residence**		
Rural	360	27.0
Urban	947	71.0
Semi urban	26	2.0
**Town of residence**		
Arusha	692	51.9
Moshi	630	47.3
Other towns	11	0.8
Total	1333	100

∞ Women partner older than men

¤ When men and women partners have similar age

### HIV prevalence among couples

Of all 1333 (100%) couples eligible for analysis, HIV prevalence among them was 13.0%, when stratified by sex, women had a higher prevalence, of 16.5%, compared to 9.5%among men. HIV prevalence by couples was as follows: 1050(79%) were concordant negative (men and women partners were all HIV negative), 63(5%) were concordant positive (men and women partners were all HIV positive) and 220(16%) were HIV sero-discordant couples. Among the HIV sero-discordant couples, the majority of HIV positive partners were women. Among the 220 couples tested with HIV sero-discordance results, 63(29%) couples had HIV positive male partners and 157(71%) had HIV positive female partners.

Table 2 shows that when the male partner was older by 10-14 years than the female partner, the proportion of men and women who were HIV positive in HIV sero-discordant union went down, compared to when men was older by 1-9 years, however there was no statistical association (χ^2^=16.21, p=0.182). Similar trends were observed when female partner was older than male by 5-9 years in comparison to when the female partner was older by 1-4 years. This association was statistically significant (χ^2^=29.64, p<0.001). Couples residing in Arusha had higher HIV prevalence rates ‘HIV sero-discordant and concordant positive’ than those of Moshi and this was statistically significant ((χ^2^=32.58, p<0.001).

**Table 2:** HIV prevalence of sero-discordance and concordance couples by socio-demographic characteristics 2005-2007

Characteristics	Couple HIV sero-status					
	Sero-discordant couples		Concordant +	Concordant -	χ^2^	P-value
	M+F-+	M-F+++	M+F+	M-F-		
	N (%)	N (%)	N (%)	N (%)		
**Men(years)**						
<19	0(0)	0(0)	0(0)	4(0.4)	**57.64**	**<0.001**
20-24	0(0)	13(8)	0(0)	153(15)		
25-29	11(18)	35(22)	7(11)	284(27)		
30-34	12(19)	31(20)	12(19)	204(19)		
35-39	16(25)	36(23)	13(21)	148(14)		
≥40	24(38)	42(27)	31(49)	257(25)		
**Total**	**63(100)**	**157(100)**	**63(100)**	**1050(100)**		
**Women(years)**						
<19	2(3)	3(2)	1(2)	112(11)	**86.45**	**<0.001**
20-24	10(16)	24(15)	6(10)	311(30)		
25-29	14(22)	45(29)	13(21)	270(26)		
30-34	17(27)	42(27)	15(24)	152(15)		
35-39	9(14)	24(15)	10(16)	82(8)		
≥40	11(18)	19(12)	18(49)	123(12)		
**Total**	**63(100)**	**157(100)**	**63(100)**	**1050(100)**		
**When women partner is older (in years)**						
1 to 4	3(75)	20(77)	1(20)	57(75)	**29.64**	**<0.001**
5 to 9	1(25)	6(23)	2(40)	18(24)		
≥10	0(0)	0(0)	2(40)	1(1)		
**Total**	**4(100)**	**26(100)**	**5(100)**	**81(100)**		
**By Socio-economic status**						
Low status	18(29)	42(8)	30(48)	350(33)	11.57	0.072
Medium status	38(60)	103(66)	31(49)	631(60)		
High status	7(11)	12(27)	2(3)	69(7)		
**Total**	**63(100)**	**157(100)**	**63(100)**	**1,050(100)**		
**By town of residence**						
Arusha	43(68)	103(65)	35(56)	509(48)	**32.58**	**<0.001**
Moshi	18(29)	53(34)	28(44)	532(51)		
Others	2(3)	1(0.6)	0(0)	9(1)		
**Total**	**63(100.0)**	**157(100.0)**	**63(100)**	**1,050(100)**		

+ HIV sero-discordant couple when men partner is HIV positive and women is negative

++ HIV sero-discordant couple when women partner is HIV positive and men is negative

### Predictors of living in HIV sero-discordance relationships

We evaluated for socio-demographic factors that predict couples being in HIV sero-discordance unions. In Table 3, we present associations between socio-demographic factors and its risks of HIV sero-discordance among couples. Compared to male partners below 35 years, their counterparts aged 35-44 years were two times more likely to either be in a HIV sero-discordance (Adjusted Odd Ratio: 2.3 95% Confidence Interval 1.7-3.2), however, as age increases their odds of infection slightly decreases (AOR: 2.2 95% CI 1.5-3.2).

**Table 3:** Predictors of being in HIV sero-discordance relationship for couples tested in Moshi and Arusha 2005-2007

Characteristics	N (%)	CRUDE OR (95% CI)	ADJUSTED OR (95% CI)
**Men age in years**			
<35	770 (58)	1	
35-44	336 (25)	2.1 (1.5-2.8)	2.3 (1.7-3.2)
≥ 45	227 (17)	1.8 (1.3-2.6)	2.2 (1.5-3.2)
Men Age in years			
< 35	1037 ( 78)	1	
35-44	216 ( 16)	2.3 (1.6-3.2)	2.6 (1.9-3.7)
≥ 45	80 ( 6)	1.1 (0.6-1.9)	1.4 (0.8-2.6)
**Age difference between women and their men partners(in years)**			
M<F	111 (8)	1.0 (0.5-2.0)	0.7 (0.3-1.7)
M=F	69 (5)	1	
1 to 5	566( 42)	0.5 (0.3-0.9)	0.7 (0.4-1.0)
6 to 10	368 (28)	0.5 (0.3-1.0)	0.9 (0.6-1.3)
≥11	219 (16)	0.6 (0.3-1.1)	-
**Socio-economic status**			
High	90 (7)	1.1 (0.6-2.0	0.8 (0.4-1.6)
Medium	803 (60)	1.0 (0.7-1.4)	0.8 (0.6-1.2)
Low	440 (33)	1	
**Distance from home to CHCT where tested (in kilometres)**			
0 to 5	862 (65)	1	
6 to 10	78 (6)	1.0 (0.5-1.7)	0.9 (0.5-1.6)
11 to 15	122 (9)	0.5 (0.3-0.9)	0.4 (0.2-0.9)
16 to 20	64 (5)	0.6 (0.3-1.2)	0.5 (0.2-1.1)
≥21	207 (16)	0.8 (0.6-1.3)	0.6 (0.4-1.1)
**Nature of area of residence**			
Rural	360 (27)	1	
Urban	947 (71)	1.3 (1.0-1.8)	2.5 (1.0-5.7)
Semi urban	26 (2)	1.1 (0.4-3.0)	0.5 (0.1-1.6)
**Town of residence**			
Arusha	692 (52)	1.9 (1.9-2.5)	2.3 (1.7-3.2)
Moshi	630 (47)	1	
Other towns	11 (1)	0.6 (0.1-2.8)	0.9 (0.1-5.3)

The bolded ORs are statistical significant

Women living with older partners by 1-4 years were less likely to be in HIV sero-discordance relationships (OR: 0.5 95% CI 0.3-09). However, after multivariate analysis, it was no longer significant (AOR: 0.7 95% CI 0.4-1.0).Couples reported to be residing in Arusha had increased risk of two times more than those residing in Moshi of being in a HIV sero-discordance relationships (AOR: 2.3 95% CI 1.7-3.2). Moreover, couples living in urban areas were more likely to be HIV sero-discordance than those living in rural areas (AOR: 2.5 95% CI 1.0-5.7). Regarding distance from their homes to the CHCT centres where they had tested, those from 11-15 kilometres from the clinic were less likely to be infected compared to those living closer to the clinic (AOR: 0.4 95% CI 0.2-0.9).

### Stratified sources of information reported by couples

We also assessed sources of the information that brought couples to come for testing in study CHCT centres. Table 4 reveals that, street banners, radio and outreach staff were the most effective strategies in passing the information about CHCT services in Arusha; tested couples reported 74%, 57% and 54% respectively of these strategies. In Moshi, friends and neighbours; which popularly were known as the ‘word of mouth’ approach were more pronounced as 55% of the couples tested as compared to 44% of couples tested in Arusha. The approach of using community outreach workers was more useful in Arusha than in Moshi. Observed differences were statistical significant (p<0.001).

**Table 4:** Stratified source of information reported by couples tested in Moshi and Arusha2005-2007

Variable	Street Banners	Posters	Word of mouth	Church/ mosque	Outreach workers	Radio	PAS+	Other sources	P-value
	N (%)	N (%)	N (%)	N (%)	N (%)	N (%)	N (%)	N (%)	
**Women age in years**									
<19	1(1)	6(6)	29(10)	4(18)	20 (9)	15(11)	13(7)	29(10)	0.015
20-24	23(33)	21(21)	73(25)	4(18)	37(17)	39(28)	52(28)	96(33)	
25-29	21(30)	28(29)	78(27)	6(27)	69(32)	18(13)	36(28)	78(27)	
30-34	14(20)	19(20)	55(19)	3(14)	43(20)	23(16)	32(12)	35(12)	
≥35	11(16)	23(23)	51(16)	5(23)	45(21)	44(29)	44(31)	55(18)	
Total	70(100)	97(100)	286(100.0)	22(100)	214(100)	139(100)	188(100)	293(100)	
**Men age in years**									
<19	0(0)	0(0)	1(0.4)	0(0)	1 (25)	0(0)	1(0.4)	1(0.3)	0.232
20-24	11(12)	13(14)	32(13)	1(5)	16 (8)	18(15)	24(11)	50(16)	
25-29	20(23)	15(16)	65(25)	6(32)	50(24)	29(24)	46(22)	98(32)	
30-34	20(23)	21(22)	48(19)	4(21)	51(24)	27(22)	27(13)	58(19)	
≥35	37(42)	45(48)	110(43)	8(42)	92(44)	48(39)	111(53)	103(34)	
Total	88(100)	94(100)	256(100.0)	19(100)	210(100)	122(100)	209(100)	310(100)	
**By Socio-economic status**									
Low	16(18)	21(22)	94(36)	7(37)	73(35)	12(10)	79(38)	96(31)	0.013
Medium	65(74)	65(70)	145(57)	11(58)	129(61)	67(55)	123(59)	187(60)	
High	7(8)	8(8)	17(7)	1(5)	8(4)	43(35)	7(3)	27(9)	
Total	88(100)	94(100)	286(100)	19(100)	210(100)	122(100)	209(100)	310(100)	
**By distance from clinic to home (in kilometres)**									
0 to 5	62(70)	69(73)	166(69)	10(53)	139(66)	76(62)	133(65)	192(62)	0.119
6 to 10	5(6)	6(6)	9(4)	1(5)	13(6)	5(4)	11(5)	26(8)	
11 to 15	5(6)	8(9)	21(8)	3(16)	19(9)	12(10)	24(11)	25(8)	
16 to 20	5(6)	2(2)	15(6)	2(11)	7(3)	11(9)	11(5)	8(3)	
≥21	11(13)	9(10)	45(18)	3(16)	32(15)	18(15)	30(14)	59(19)	
Total	86(100)	94(100)	256(100)	19(100)	210(100)	122(100)	209(100)	310(100)	
**Nature of place of residence**									
Rural	20(23)	22(23)	74(29)	8(42)	50(24)	38(32)	56(27)	84(27)	0.778
Urban	67(76)	72(77)	177(69)	11(58)	155(74)	82(67)	148(71)	218(70)	
Other	1(1)	0(0)	5(2)	0(0)	5(2)	2(1)	5(2)	8(3)	
Total	88(100)	94(100)	256(100)	19(100)	210(100)	122(100)	209(100)	310(100)	
**Town of residence**									
Arusha	65(74)	31(33)	112(44)	12(63)	114(54)	70(57)	39(19)	235(76)	<0.001
Moshi	23(26)	62(66)	140(55)	7(37)	95(45)	51(42)	169(80)	72(23)	
Others	0(0)	1(1)	4(1)	0(0)	1(0.4)	1(1)	1(0.4)	3(1)	
Total	88(100)	94(100)	256(100)	19(100)	210(100)	122(100)	209(100)	310(100)	

+ Public address system whereby a car was loaded with speakers and mobilize couples to go for testing

Regarding age and source of information, immense proportion of both men 39(28%) and women 18 (15%) aged 20-24 years were more likely to report radio as a source of information. These observations were statistically significant for the women (p=0.015) but not the men partners (p=0.232). However, a public address system was more prominent among middle and low socio-economic status couples by 123(59%) and 79(38%) respectively. Radio was a more useful means of communicating about CHCT among couples of middle and high socio-economic status than low socio-economic status couples (p=0.013).

Of all couples who reported public address system 169 (80%) of these couples were residing in Moshi and 39(19%) were from Arusha. All couples who reported radio 70(57%) were from Arusha and 51(42%) from Moshi, only 1% were from neighbouring regions (p<0.001).

### Sources of information that brought HIV sero-discordant couples for testing

We grouped together all HIV concordant positive and HIV concordant negative to form one group intentionally to compare the promotion strategies that brought HIV sero-discordant to be tested. Table 5, shows that among all sources of information, community outreach workers/clinic staff and ***‘word of mouth’*** were two times more likely to be reported by couples in HIV sero-discordance relationships than those concordant positive and negative couples (OR: 1.6, 95% CI 1.1-2.5). Following adjustment for all other demographic factors this promotion strategy maintained its significance (AOR: 1.8 95% CI 1.2-2.8).

**Table 5:** Source of information that reported by HIV sero-discordant couples who received CHCT services in Moshi and Arusha

	CRUDE OR (95% CI)	ADJUSTED OR (95% CI)
Banners	0.7 (0.4-1.4)	0.7 (0.4-1.4)
Fliers/Leaflets	1.4 (0.5-3.7)	1.8 (0.7-4.6)
Posters	0.3 (0.1-0.7)	0.4 (0.2-1.0)
Friends/neighbours	1.2 (0.8-1.7)	1.4 (0.9-2.1)
Church/mosque	1.0 (0.3-3.1)	1.0 (0.3-3.4)
OW/Clinic staff	1.6 (1.1-2.5)	1.8 (1.2-2.8)
Radio	0.9 (0.5-1.6)	1.0 (0.6-1.8)
Public Address System	0.7 (0.4-1.2)	0.9 (0.5-1.5)
Other sources	1	1

The bolded ORs are statistical significant

Besides that, fliers, and word of mouth were also much likely to be mentioned by couples who report the same source of information although they were not statistically significant (Table 5).

## Discussion

We implemented promotion campaigns for about 2 years purposely to understand its impact in increasing uptake of CHCT services, prevalence of HIV and predictors of infection among couples tested in our centres. To the best of our knowledge, this was first and only study in Tanzania to involve massive promotions of exclusive CHCT services especially in the northern part of the country.

### Prevalence of HIV sero-discordance

In this study, we found that the prevalence of HIV sero-discordance was as higher as 16%. This finding further confirms that the proportion of HIV sero-discordant couples is very high than HIV concordant positive couples in SSA. Similar findings are confirmed in studies conducted in Tanzania and elsewhere which reported the sero-discordance prevalence ranging from 5% to75% [5–7, 9, 12, 19, 21, 27, 44–47]. The prevalence of HIV sero-discordance in our study could be higher than in national representative population-based surveys due to self-selection of couples who presented to utilize CHCT services and the objective of the main study in recruiting HIV sero-discordant couples only. However, our study continues to substantiate urgent need for developing more interventions that will reverse HIV infection including treatment as prevention among HIV sero-discordant couples in sub-Saharan Africa and Tanzania in particularly.

### Distribution of HIV sero-discordant couples

Our study established that women were more likely to be HIV positive in HIV sero-discordance relationships as compared to their men counterparts. This finding coincides with a multi-country study that analysed national representative population surveys data from eleven sub-Saharan Africa countries [6, 48]. Moreover, Mujugira et al, in their multi-country study in Eastern and Southern African countries, found that of the 3,408 HIV sero-discordant couples enrolled, 67% of the HIV infected partners were women [49]. Apart from their biological susceptibility to HIV infection per sexual act, young women in Tanzania and other SSA countries also initiate sex with older partners earlier than their male counterparts do. In addition, due to social desirability bias, women in stable relationships practice concurrent partnerships more than reported [50].

Other ways of transmission include infection while caring for AIDS patients [1, 9, 11, 12, 51]. However, this is in contrast with recent findings from a study conducted by Eyawo et al which analysed data from 14 SSA countries, the study showed that women account for 47% of infected partners in HIV sero-discodance relationships [10]. Majority of our study participants consisted of women partners aged 18-29 years, the age group that considered being at high risk of HIV infection. Therefore, our study findings suggest that intensification of HIV prevention programmes that target young girls and adult women in community is vital.

### Predictors of HIV infection

Moreover, we found that HIV sero-discordance was strongly associated with living in urban areas as compared to rural. Besides, HIV surveys carried out in Tanzania and elsewhere established that infected couples were more likely to live in urban areas than rural [12, 52]. This high prevalence among couples living in urban areas especially in Arusha and Moshi, could be attributed to other causal factors, for instance, the high risk behaviours, like having multiple sexual partners with low use of condom. Others are due to inappropriate use of condoms associated with high consumption of alcohol before sex [12, 48, 53, 54]. Our findings could be biased by the large number of urban dwellers who showed up for testing than those from rural areas since all our centres were located in towns. While, we sometimes promoted CHCT in rural areas and conducted mobile VCT there, we were not able to measure HIV prevalence or monitor uptake of CHCT services in those localities. We therefore suggest more studies on CHCT promotion that target rural residents to be conducted as HIV and AIDS affect both rural and urban residents.

### Age difference among partners as risk for HIV infection

This study did not find any associations between risk of infection and age differences among partners in sero-discordance relationships. This finding is in line with the other study carried out in Uganda among couples who presented for VCT [55]. However, the the finding is inconsistent with those of previous studies done in Uganda and Tanzania which established that women living with older men partners by more than 12 years were more likely to be in a HIV positive union(either sero-discordance or concordance positive). The reason given was that the older men partners were more likely to be infected long before entering into the new union with their current women partners. Besides, women are less likely to discuss about safer sex because of their economic dependence on their men partners [13, 34] [35, 56]. This inconsistency could be due to differences in the research designs, nature of population and sample size used by our study and theirs.

### Effective CHTC promotion strategy mobilising HIV sero-discodance couples to come for testing

Dissemination of CHCT messages through community outreach workers and friends proved to be feasible in these settings among couples living in urban areas. Previous reports from Uganda uncovered that only a small proportion of women, particularly those residing in urban areas and with at least formal education, were able to initiate and discuss with their men partners about condom use and contraceptives [57]. Discussions on other sexual issues, like HIV testing, were more likely to cause marital conflicts whereby men partners might feel that they are suspected to be infected or unfaithful. Therefore, in this situation having someone from the outside who will discuss with both partners about knowing their HIV status was very crucial [36, 57, 58]. In our study, most of HIV sero-discodance couples were more likely to have been invited by community outreach workers to come for testing. This finding may possibly be biased by the core interest of the main study that was to recruit HIV sero-discordance couples only. Therefore, some people living with HIV and AIDS were identified from different organizations/groups and others community members who wanted to test individually in our study CHCT centres and other collaborating VCT centres were persuaded to bring their partners so that they can be tested as a couple. Nonetheless, using community outreach workers was a labour intensive and time consuming strategy, since long discussions on HIV sero-discordance status were initiated due to poor knowledge about the subject in these communities like elsewhere in Sub Saharan Africa [21, 41]. Thus, other promotion strategies like information, education and communication materials should be also used to supplement community outreach strategy.

Among all couples that tested, about 1% reported to be residing in other towns outside the study catchments area. This gives an impression that because of trickle down of information from sources such as the radio reaching beyond the study's target population probably gave opportunity for them to come over and get tested. The other impression could be probably unavailability of CHCT services or stigma and lack of trust towards healthcare workers in their localities.

#### Limitation of the study

While our study maintained its internal validity through use of standardized HIV tests and pre-tested clinic checklist, nevertheless its findings should be cautiously generalized because of self-selection of couples who persuaded and presented for testing. In most cases, we relied on self-reporting of couples if they were cohabiting and even other socio-demographic information. The situation and nature of the study did not allow us to verify the information given. Self-reporting is prone to bias which can lead to underreporting or over reporting of one's information. Lastly, we were not able to enquire more information on other factors that could have been associated with HIV sero-discordance. Since the study did not collect information such as condom use, duration of union, if a men partner was circumcised, the level of alcohol use, concurrent partnerships, number of children and desire to have children. We also did not have information on the presence of other sexual transmitted infections, or if an infected partners were already on ART.

## Conclusion

Promotion campaigns were effective in increasing utilization of CHCT services, which lead to our understating of HIV sero-discordance prevalence and its predictors among cohabiting couples in Moshi and Arusha. Our study findings add more on studies that highlighted HIV prevalence and predictors of risks factors of infection among couples. Since, cohabiting couples represents very important population segment that responsible for reproduction and caring of children. Therefore, developing more CHCT centres equipped with trained counsellors where motivated couples will go for testing is critical to curb infection through wide access of treatment-as-prevention strategy. We strongly suggest use of multiple channels of communication in promoting CHCT services that discourage ***test by proxy*** and educate communities about HIV sero-discordance notion.
